# Parental Involvement in Child Anxiety and Depression: Exploring the Impact of Delivery Format on Modifiable Parental Factors

**DOI:** 10.1007/s10578-025-01814-6

**Published:** 2025-02-13

**Authors:** Kristin Ytreland, Elisabeth Valmyr Bania, Stian Lydersen, Anne Mari Sund, Simon-Peter Neumer, Frode Adolfsen, Kristin Dagmar Martinsen, Lene-Mari Potulski Rasmussen, Jo Magne Ingul

**Affiliations:** 1 Regional Centre for Child and Youth Mental Health and Child Welfare, Central Norway, Faculty of Medicine and Health Science, Department of Mental Health, Norwegian University of Science and Technology (NTNU), Postbox 8905 MTFS, 7491 Trondheim, Norway; 2 Regional Centre for Child and Adolescent Mental Health, Eastern and Southern Norway (RBUP Øst og Sør), Oslo, Norway; 3 Regional Centre for Child and Youth Mental Health and Child Welfare – Northern Norway, Faculty of Health Sciences, UiT - The Arctic University of Norway, Tromsø, Norway; 4 Department of Psychology, University of Oslo, Oslo, Norway

**Keywords:** Anxiety, Depression, Parental involvement, Parenting, Prevention, Children

## Abstract

Prior research has linked parental factors such as parent psychopathology, family functioning, parenting style, and parental practices to child anxiety and depression. Parents are often involved in interventions for these disorders. However, previous research suggests that this does not always add to the effect of child treatment alone. Furthermore, little research report changes in known parental risk and protective factors. As part of the ECHO-trial, we examined two delivery formats of parental involvement in the *Emotion* intervention, an indicated school-based cognitive behavioral therapy (CBT) program to prevent child anxiety and depression. Parents received either five parent group sessions or a brochure, while their children attended group sessions. Parents (*N* = 1028) completed our online survey at baseline, post-intervention, and/or 12-month follow-up. On average, parents showed small improvements in anxiety and depression symptoms and parental practices over time. There was no difference in parental factors between parents in the group sessions and the brochure condition.

The trial was preregistered at https://www.clinicaltrials.gov/ (NCT04263558), in February 2020.

## Introduction

Left unaddressed, anxious and depressive problems in children can develop into disorders, impacting various life aspects and prove costly for the individual, family, and society [36]. Therefore, early intervention, through evidence-based programs, is crucial for preventing these disorders and enhancing quality of life [21]. Given the high occurrence of these disorders [20, 34] and the role of modifiable parental factors in their development and persistence [39], parents are often included in prevention and treatment efforts [10, 40]. However, the impact of such inclusion remains unclear [4, 5], indicating that it does not add to the effect of child interventions. This study investigates whether known parental factors: parents’ own symptoms, family functioning, parenting styles and parental practices, change through participation in a school-based preventive intervention for child anxiety and depression, and whether delivery format, i.e. five parent sessions or a parent brochure, affects parental outcomes.

### Influences on the Development and Maintenance of Child Anxiety and Depression

Children’s internalizing problems stem from both genetic and environmental factors [29, 37]. As primary caregivers, parents significantly influence their children’s development. An imbalance of risk factors over protective factors may result in anxiety or depression. Schleider and Weisz’s [29] tridirectional model underlines the importance of the family process for the development of internalizing problems on three levels: parent-level factors such as parent symptomatology; family-level factors such as family functioning; and dyad-level factors such as parenting styles or practices. These factors are hypothesized to affect cognitive styles and attentional bias in the child, which can influence the development of internalizing problems. In turn, children’s internalizing problems, such as attention biases or withdrawal, may affect family processes on all three levels.

### Parental Factors Associated With Child Anxiety and Depression

A meta-analysis by Yap and Jorm [39] identified several parental factors linked to child internalizing problems in previous longitudinal research, supporting Schleider and Weisz’s model (2017). Risk factors were abuse, aversiveness, interparental conflict, and over-involvement. Parental *warmth* was identified as a protective factor.

Children of parents with anxiety or depression are more than twice as likely to develop anxiety and depression themselves, compared to children of parents without these disorders [9, 14]. Parent psychopathology may also hinder children’s treatment effect. In a cognitive behavioral therapy (CBT) study of anxiety in 8- to 12-year-olds, paternal rejection, anxious and depressive symptoms, and more surprisingly, maternal warmth was associated with poorer outcomes [15]. The authors suggested that fathers’ depressed mood might have led to more rejecting behavior towards their children, affecting their reports of child anxiety. A Norwegian study on parent and child depressive symptoms indicated that fathers’ absence of depression could buffer against the negative impact of maternal depression [8].

Family functioning also appears to affect child anxiety and depression. In an Australian study of a universal program targeting internalizing symptoms in children aged 9–11 years, Kennedy et al. [13] reported no difference in the effect of the intervention or control group on child symptoms. However, child-reported family functioning predicted child internalizing symptoms six months later.

In a meta-analysis, Pinquart [25] examined the association between child and adolescent internalizing disorders and parenting style dimensions. He reported that parental warmth, behavioral control, and autonomy-granting parenting styles might serve as protective factors for offspring internalizing symptoms. On the other hand, parenting characterized by harsh and psychological control and authoritarianism was associated with poorer child outcomes.

Research has also linked parental practices to child anxiety and depression. In an Australian RCT [30], parents who received an online intervention targeting parental risk and protective factors for child anxiety and depression demonstrated further improvement in the Parenting to Reduce Child Anxiety and Depression Scale (PaRCADS) [31], one year later, compared to parents in the control group. This could indicate that parental practices can be modified through targeted parent interventions to enhance parental factors related to child anxiety and depression.

Unlike genetic inheritability and socioeconomic status, behavioral parental factors are potentially modifiable. Studies have demonstrated that these factors significantly contribute to the development and maintenance of child anxiety and depression. However, the effects of including parents in interventions are mixed [4, 5]. Interestingly, in a meta-analysis of child and adolescent anxiety, Jewell et al. [11] reported significant treatment effects of targeting parents as the main client compared to waitlist controls. However, they found no difference between parent-only and child-only treatments. This implies that while parental involvement is effective, the way it has been done does not add incremental effects, compared to child-only treatment. This could be because parental components often mirror what children learn in their sessions, for example, the importance of exposure and ensuring follow-up at home. Previous research has not necessarily set out to modify parental risk and protective factors [3]. Hence, the similarity in content with children’s sessions, might not provide additional benefits.

### The ECHO-Trial

This study aims to investigate whether key parental factors associated with the development and maintenance of anxiety and depression change as a result of participating in parent sessions or reading a brochure, as part of the larger ECHO-trial [22]. The ECHO-trial is a randomized factorial trial with three factors, each with two levels, resulting in 8 different experimental conditions. The trial seeks to optimize a transdiagnostic, preventive, school-based group CBT intervention, *Emotion,* targeting children aged 8–12 years with elevated levels of anxiety and/or depressive symptoms [18].

Due to the lack of evidence to support the additional effect of parental involvement in child anxiety and depression interventions, we compared a less resource-demanding delivery format; a psychoeducational brochure, to the original parental involvement component of *Emotion*; five parent group sessions. Both children and their parents in the group session condition and brochure condition reported equivalently reduced child symptoms between baseline and post-intervention [16]. In other words, there was no statistically significant difference in the effects on children’s symptoms between families who received the two formats of delivery.

### Study Objectives

Most existing studies on parental involvement are treatment studies and vary methodologically in terms of sample size, duration, intensity, extent of involvement and outcome variables [4, 5]. In a review and meta-analysis, Werner-Seidler et al. [38] report small effect sizes for school-based prevention programs in reducing child anxiety and depression, with greater effect sizes for depression in studies with indicated samples (i.e. children at risk or with elevated symptoms of depression). However, previous studies on school-based prevention programs primarily focus on changes in the child, not the parent.

Based on the knowledge that parental factors are linked to the development and maintenance of these problems, modifying parental factors could potentially strengthen the effects of the intervention both in the short and long-term. One plausible explanation for the limited effectiveness of parental involvement could be that parental factors are not sufficiently targeted [3]. Therefore, we wanted to investigate whether the delivery format of the parent component in *Emotion* predicted the level of change in modifiable parental factors linked to child anxiety and depression: parent symptomatology; family functioning; parenting style; and parental practices. Understanding these changes can guide future practice and research on parental involvement in interventions for child anxiety and depression problems.

Given the evidence that parents are related to the development and persistence of anxiety and depression [39] and the nil findings of Lisøy et al. [16], that children, regardless of delivery format of the parental component in ECHO, reported significantly less anxiety and depression scores post intervention, we hypothesized no difference in parental factors: parent’s anxiety and depression symptoms; family functioning; parenting style; and parental practices between the two groups post-intervention and at 12-month follow-up. We also wanted to explore whether parents participating in the ECHO-trial reported changes in these parental factors during and after the intervention.

## Methods

### Study Design

This study is part of a cluster randomized factorial trial, ECHO, that aimed to optimize the preventive intervention, *Emotion*, by providing the best possible outcome within the constraints imposed by the need for efficiency and economy faced by service providers. We compared three potentially optimized components, to the original intervention:

1) Intervention Delivery Format: Original, 16 sessions vs. blended format where eight alternating child-sessions were prerecorded online, and eight were in the original face-to-face group format.

2) Parental Involvement delivery format: Original, five group sessions vs. parent brochure.

3) Measurement Feedback System (MFS): Original, no feedback vs. a mobile app where children reported weekly on their development.

These three components were set to two levels, "original” or “optimized”, resulting in eight experimental conditions. In the present paper, we studied whether factor two, the delivery format of parental involvement, predicted the change in parental factors linked to child anxiety and depression. Thus, this factor is the focus in the current study. For more information about the ECHO study design, see Neumer et al. [22].

### Intervention

The goal of the *Emotion* intervention is to prevent 8–12 year old children’s anxiety and/or depressive problems from developing into a diagnosis. The original RCT of the intervention showed positive effects on child anxiety and depression outcomes [19]. However, group leaders and their service leaders provided feedback emphasizing time constraints and services’ workload as barriers to the implementation of *Emotion* [26]. Thus, the *Emotion* intervention was shortened from 20 to 16 group sessions for children, and from seven to five group sessions for parents over eight weeks. Group leaders are personnel from first-line services, who receive formal training, and regular supervision.

### Parental Involvement Delivery Format

**Fig. 1:**
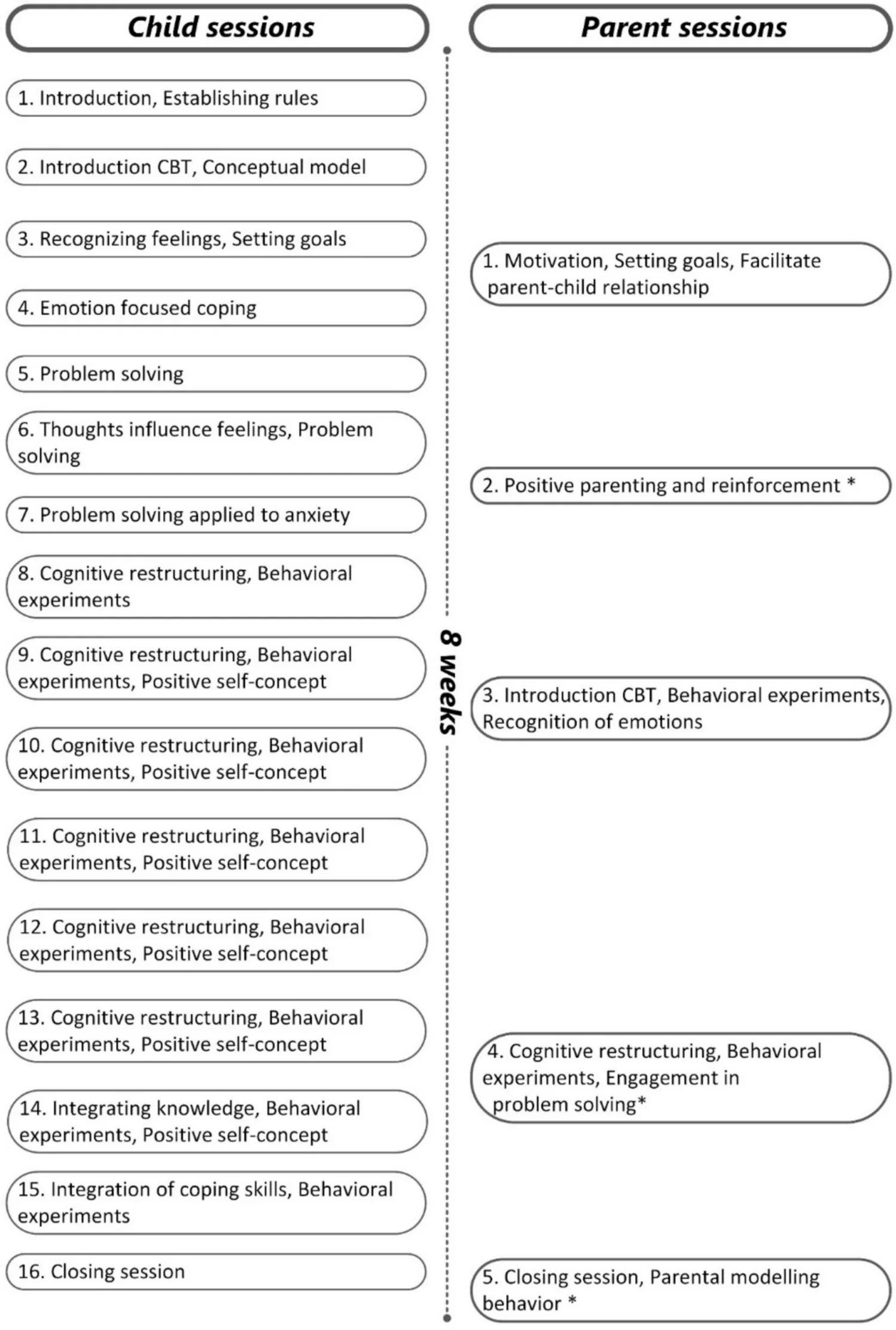
Overview of topics in child and parent group sessions in the ECHO-trial. Children attended parent sessions 2, 4 and 5, marked with an asterisk (*)

Parents in the original parental involvement condition were invited to participate in five parent group sessions, receiving the *Emotion* parent workbook with exercises for both sessions and home. Children attended three of these sessions and those may be referred to as family sessions. These sessions offer families the chance to discuss the intervention and complete exercises together in a supervised setting. See Fig. 1 for session topics in both parent and child group sessions.

Parents in the brochure condition received a 20-page psychoeducational brochure^1^ based on the parent sessions’ content and workbook. While similar in content, the brochure briefly explained the main strategies children would learn, equipping parents to support their child. Unlike group sessions, the brochure provided general information, where parent-sessions also provided tailored examples of strategies and homework tasks. Both groups attended an initial meeting for practical information about the intervention. This meeting was distinct from the parent sessions, which included practice, discussions and relevant examples. Parents in the brochure condition were given the brochure and informed it contained useful information to support their child, but were not explicitly instructed to read it. After this meeting, there was no further organized contact between parents and group leaders in the brochure condition.

In the parental component of *Emotion*, emphasis is placed on helping parents recognize and address their children’s sad and anxious feelings. This included psychoeducation on anxiety and sadness, and explaining the strategies children would learn in their group sessions to enhance parental support. The specific CBT concepts covered are:

- **Behavioral Activation:** Encouraging positive parenting to improve mood and reduce depressive symptoms through positive reinforcement.
- **Emotional Awareness:** Teaching parents to recognize and name emotions and model healthy emotional responses.
- **Coping Skills Training:** Guiding parents to help children apply effective coping strategies for managing anxiety and depression.
- **Problem Solving:** Highlighting techniques to identify challenges and generate solutions.
- **Cognitive Restructuring:** Assisting parents in helping children challenge distorted thoughts.
- **Behavioral Experiments:** Encouraging empirical testing of beliefs to foster healthier thought patterns.
- **Positive Reinforcement:** Promoting desirable behaviors through praise and rewards.

### Procedure and Participants

**Fig. 2:**
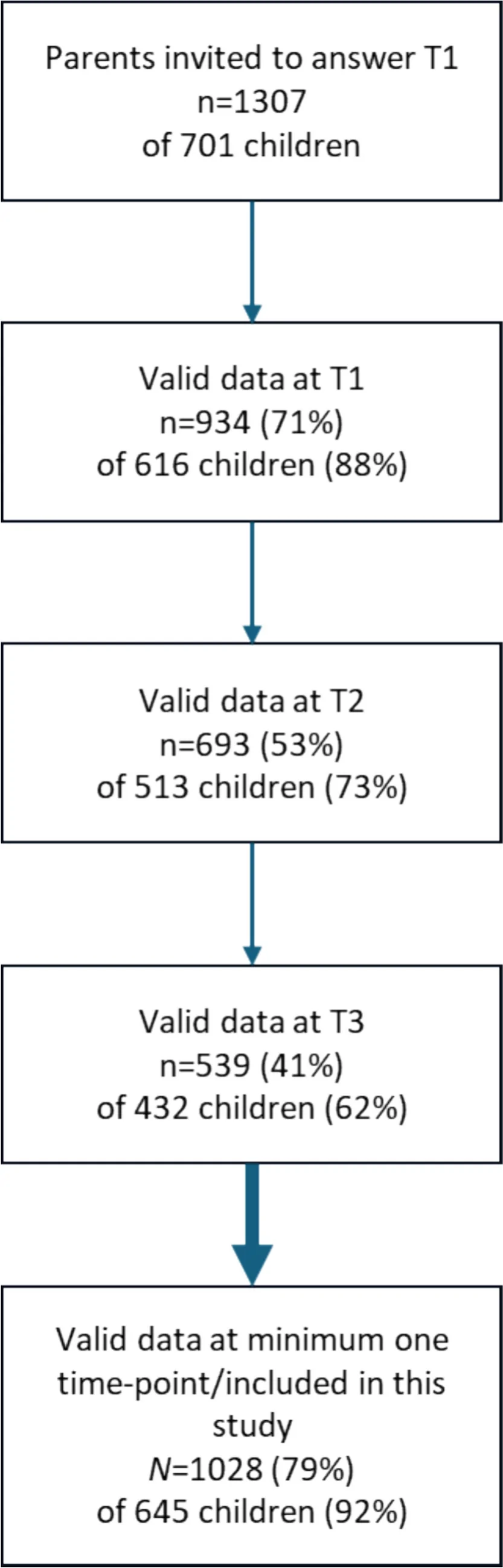
Participant flow, number of parents and children included in this study

In the ECHO-trial, participants were recruited from 58 schools across Norway between 2020 and 2023. Schools were randomized to experimental conditions, before children and parents were invited to participate, with standardized information material about the project. Children aged 8–12 years with anxiety or depression symptoms and their parents were encouraged to participate. Interested children with parental consent were screened online for anxiety and depression symptoms using the Multidimensional Anxiety Scale for Children (MASC; [17]) and the Moods and Feeling Questionnaire short form (SMFQ,[1]). Up to seven children from each school, who scored ≥ 1 standard deviation above the expected population mean, based on previous studies of population samples, on either measure, were invited to participate in the intervention. Cut off values were ≥ 61 points for girls, ≥ 54 points for boys on the MASC scale based on Ólason et al. [23], and ≥ 7 points for all participants on the SMFQ scale based on Rhew et al. [27]. Parents (*n* = 1307 of the enrolled children (*n* = 701) were invited to answer the questionnaire. The participants included in this paper were parents of children who participated in the intervention and responded to parent questionnaires on one or more occasions: before the intervention (T1), immediately after the intervention (T2), and at 12-month follow-up (T3). The participant flow is presented in Fig. 2. The study was approved by the Regional Committee for Medical and Health Research Ethics—Southeast Norway (2019/1198) and The Norwegian Agency for Shared Services in Education and Research (152745).

### Measures

Child age was collected via the consent form, and parent demographic information was collected the first time the parent answered the questionnaire. Instruments used in this paper are described in Table 1. To reduce the length of the questionnaire, we used only five out of ten subscales of the PaRCADS(N) (Parenting to Reduce Child Anxiety and Depression Scale—Norwegian version) in this study.

**Table 1:** An overview of the instruments/subscales and the parental factors they measure, with internal consistency values from previous and the current study, reported in Cronbach´s Alpha (α) and McDonald’s Omega (ω) to indicate the psychometric properties of each instrument/subscale

Instrument, subscales (items) reference	Parental factor Description of instrument/subscale	Scoring *Example item*	Reported psychometric properties reference	Current psychometric properties
HSCL-10 (10) [6]	Parent’s symptoms Psychological distress, anxiety and depression symptoms	0 (not at all) to 3 (extremely) *Suddenly scared for no reason*	α = 0.88 [35]	ω = 0.88
FAD-GF (12) [7]	Family functioning Families healthy and unhealthy functioning	1 (strongly agree) to 4 (strongly disagree) *Planning family activities is difficult because we misunderstand each other. (FP)*	α = 0.87 [41]	ω = 0.86
PBI (16) [12, 24]	Parenting style	1 (fits very well) to 4 (fits very poorly)		
Warm (7)	Positive emotions and empathetic communication	*Spoke to him/her with warm and friendly voice*	α = 0.77 [28]	ω = 0.78
Protective (5)	Control and infantilizing behavior	*Was overprotective*	α = 0.69 [28]	ω = 0.66
Authoritarian (4)	Restrictive and directing behavior	*Lets child decide things for self. (FP)*	α = 0.51 [28]	ω = 0.55
PaRCADS(N) (78) [31, 41]	Parental practices	0 (almost never/very unlikely) to 4 (almost always/very likely)		
Rules & consequences (9)	Setting clear and consistent boundaries, adaptive use of discipline and rewards	*I have set specific, defined rules for his/her behavior*	ω = 0.69 [41]	ω = 0.68
Health habits (7)	Encouraging good health habits, e.g. sleep hygiene and exercise	*I practice good health habits (i.e. healthy diet, regular exercise, good sleep habits)*	ω = 0.57 [41]	ω = 0.62
Managing emotions (7)	Helping their child regulate emotions and increase resilience	*I encourage my child to have an accepting and realistic view of him/her self*	ω = 0.62 [41]	ω = 0.53
Setting goals & dealing with problems (8)	Helping their child develop perseverance, goal-setting and problem-solving skills	*I help my child learn from his/her mistakes*	ω = 0.73 [41]	ω = 0.72
Dealing with negative emotions (10)	Helping children manage and cope with anxious or sad emotions before they become too overwhelming	*I encourage my child to face situations that he/she is afraid of*	ω = 0.60 [41]	ω = 0.63

*HSCL-10* Hopkins symptoms checklist -10 item version, *FAD-GF* Family assessment Device–General Functioning subscale, *PBI* Parental bonding instrument, *PaRCADS(N)* Parenting to Reduce Child Anxiety and Depression Scale (Norwegian version), *FP* False positive items that were reverse coded

### Statistical Analyses

We used linear mixed effects models with the modifiable parental factors, one at a time, as the dependent variable. Parental involvement and measurement time and their interaction were included as categorical covariates. Data were available from both parents for 60% of children (n = 393). We included the random effects of parents nested within child-ID, nested within school. Linear mixed models include participants with complete and partly missing data, and the results are unbiased if data are missing at random (MAR). Analyses were carried out unadjusted and adjusted for parental age and sex to control for potential confounders. Additionally, to see whether the level of parental adherence, i.e. attendance in sessions and reading the brochure affected the results, we performed the same analyses as described above including only a subsample of parents with high levels of adherence (n = 443). This sample consisted of parents from the parent sessions condition where group-leaders had reported they attended at least three out of the five sessions (n = 258), and parents who reported they had read the brochure during the intervention (n = 185). Lastly, we used linear mixed effect models as above, without parental involvement, to study the average effect of time. We used the robust variance estimator to allow for skewed distribution of the dependent variables. Finally, *p*-values were corrected with Benjamin Hochberg’s False Discovery Rate procedure in R. Analyses were carried out in IBM SPSS v.29 and Stata v.17.

## Results

The demographic information is presented in Table 2. Our sample consisted of 1028 parents of 645 children aged 8.6–12.8 years, who participated in the ECHO-trial. Nine percent were currently not working, and the most frequent reasons were “unable to work” (3%) and “under work assessment allowance” (2%). Sixty-seven percent of the participants had higher education from university or college, a larger proportion than the same age group in the general Norwegian population (50%) [33]. In the parent sessions condition, children (*n* = 438) were represented by at least one parent in 79% of the sessions. Both parents were invited, thus some parent pairs shared the sessions between them. On average mothers attended 3.5 sessions (*SD* = 1.4) fathers attended 2.5 sessions (*SD* = 1.6). At the post-intervention survey, parents in the brochure condition (*n* = 590), were asked about the brochure. Among the 391 respondents, 82% (*n* = 320) reported that they had received the brochure, of which 58% (*n* = 185) reported that they read it during the intervention (64% of mothers, and 47% of fathers) and 38% (*n* = 123) had turned its pages (36% of mothers, and 45% of fathers), indicating that they had opened the brochure and read some of its content, but not read it thoroughly.

**Table 2:** Demographic information about the participants (N = 1028)

Parent characteristics	*n*	%
Relationship to child		
Mother	616	60%
Father	400	39%
Other	12	1%
Foster parent	10	1%
Stepparent	2	0%
Age (mean years, *SD*)	41.9 (6.1)	
Birthplace (*n* = 1026)		
Norway	913	89%
Employment		
Full time	823	80%
Part-time	116	11%
Currently not working	89	9%
Completed education		
Lower secondary school	162	16%
Upper secondary school	180	18%
University/College ≤ 4 years	335	33%
University/College > 4 years	351	34%
Family gross income*		
> 1 000 000 NOK	478	47%
801 000 to 1 000 000 NOK	211	21%
501 000 to 800 000 NOK	211	21%
351 000 to 500 000 NOK	83	8%
201 000 to 350 000 NOK	38	4%
< 200 000 NOK	7	1%
Parents evaluation of family income		
Very good	133	13%
Good	611	59%
Mediocre	259	25%
Poor	20	2%
Very poor	5	1%
Household		
Both parents	709	69%
Shared custody	187	18%
Only mother	59	6%
Only father	4	0%
Parent and stepparent	56	5%
Foster parent(s)	11	1%
Other	2	0%
Child		
Daughter	633	62%
Son	395	38%
Child Age (mean years, *SD*)	10.5 (0.7)	

^*^1 NOK = approximately 0.1 Euro in 2022

### Between Group Differences

Table 3 shows descriptive statistics with sample ranges for the outcome variables and the estimated effect of the brochure condition versus the parent sessions condition. The estimates were small and statistically nonsignificant for all outcome variables and time points. Results from models adjusted for parents’ education level, age and sex, and sensitivity analyses from the subsample of adhering parents were substantially the same (data are not shown).

**Table 3:** Descriptive statistics of parental factors for the two subsamples in the brochure and parent sessions condition at baseline (T1), post-intervention (T2) and 12-month follow-up (T3)

Dependent variables	Range	Descriptive statistics				Between group differences		
		Parent sessions		Brochure				
		*n*	*M* (*SD*)	*n*	*M* (*SD*)	Est	95% CI	*p*-value
Parent’s symptoms, HSCL 10^a^	0–28							
T1		394	4.7 (4.6)	527	5.1 (4.7)			
T2		273	4.7 (4.9)	400	4.5 (4.5)	− 0.48	[− 0.89 to − 0.07]	.17
T3		229	4.7 (4.6)	302	4.3 (4.3)	− 0.49	[− 1.11 to 0.14]	.51
Family functioning, FAD-GF^a^	12–37							
T1		394	19.4 (4.9)	527	19.3 (4.6)			
T2		273	19.5 (4.6)	401	19.3 (4.7)	− 0.04	[− 0.50 to 0.41]	.99
T3		229	19.3 (4.7)	303	19.9 (4.6)	0.69	[1.15 to 1.23]	.17
Parenting styles PBI								
Warm^b^	7–18							
T1		394	10.5 (2.4)	529	10.6 (2.4)			
T2		274	10.4 (2.3)	401	10.5 (2.2)	0.06	[− 0.21 to 0.33]	.90
T3		229	10.2 (2.3)	304	10.7 (2.4)	0.31	[− 0.07 to 0.69]	.51
Protective^a^	9–20							
T1		394	16.9 (2.0)	529	16.9 (1.9)			
T2		274	17.2 (1.8)	401	17.2 (1.9)	− 0.01	[− 0.21 to 0.20]	.99
T3		229	17.3 (1.8)	304	17.2 (1.9)	− 0.11	[− 0.37 to 0.16]	.79
Authoritarian^a^	7–16							
T1		394	12.5 (1.5)	529	12.4 (1.5)			
T2		274	12.6 (1.5)	401	12.6 (1.5)	0.07	[− 0.13 to 0.27]	.79
T3		229	12.8 (1.4)	304	12.7 (1.5)	− 0.07	[− 0.30 to 0.16]	.79
Parental Practices PaRCADS(N)^b^								
Rules & consequences^b^	12–36							
T1		397	24.6 (3.7)	532	24.2 (3.9)			
T2		279	24.8 (3.8)	406	24.7 (3.8)	− 0.00	[− 0.57 to 0.56]	.99
T3		229	25.1 (3.6)	307	24.0 (3.7)	− 0.62	[− 1.17 to − 0.07]	.17
Health habits^b^	4–24							
T1		396	17.5 (3.0)	531	17.5 (3.3)			
T2		278	17.3 (3.0)	403	17.4 (3.0)	− 0.01	[− 0.34 to 0.33]	.99
T3		229	17.1 (3.1)	305	16.9 (3.1)	− 0.23	[− 0.70 to 0.23]	.79
Managing emotions^b^	8–28							
T1		398	19.3 (3.4)	536	19.3 (3.5)			
T2		283	20.1 (3.4)	410	20.1 (3.2)	− 0.00	[− 0.35 to 0.35]	.99
T3		230	20.2 (3.1)	309	20.0 (3.4)	− 0.15	[− 0.66 to 0.35]	.79
Setting goals & dealing with problems^b^	13–32							
T1		398	23.8 (4.0)	534	23.6 (3.8)			
T2		281	24.3 (3.8)	408	24.4 (3.4)	0.17	[− 0.26 to 0.61]	.79
T3		230	24.1 (3.7)	309	23.7 (3.7)	− 0.17	[− 0.62 to 0.29]	.79
Dealing with negative emotions^b^	16–40							
T1		397	28.4 (3.7)	532	28.3 (3.9)			
T2		280	29.5 (3.8)	407	29.3 (3.7)	− 0.25	[− 0.69 to 0.20]	.79
T3		229	29.5 (3.9)	308	29.0 (4.1)	− 0.30	[− 0.85 to 0.24]	.79

Between group differences for the brochure versus parent sessions condition are coefficients for the interaction between delivery format and time from linear mixed models. *p*-values were Benjamini Hochberg adjusted for 20 hypotheses

^a^Higher values = less adaptive parenting, ^b^Lower values = more adaptive parenting

*HSCL-10* Hopkins Symptoms Checklist -10 item version, *FAD-GF* Family Assessment Device—General Functioning subscale, *PBI* Parental Bonding Instrument, *PaRCADS(N)* Parenting to reduce child anxiety and depression scale (Norwegian version)

### Changes in Parental Factors Linked to Child Anxiety and Depression

Descriptive statistics and *p*-values from unadjusted linear mixed effects models on parental factors linked to child anxiety and depression are presented in Table 4. Overall, parents reported a statistically significant positive change between baseline and post-intervention on symptoms (HSCL 10) and parental practices as measured by PaRCADS(N) subscales: *Managing emotions*, *Setting goals & dealing with problems* and *Dealing with negative emotions*. The positive effects of time on parents’ symptoms, *Managing emotions* and *Dealing with negative emotions* were stable at 12-month follow-up. Parents reported a statistically significant increase in *Protective* and *Authoritarian* scores, this effect was stable at 12-month follow-up. Parents also showed a statistically significant decline in *Health habits* scores from baseline to 12-month follow-up.

**Table 4:** Descriptive statistics for the total sample at baseline (T1), post-intervention (T2) and 12-month follow-up (T3)

Dependent variables	Total sample *M* (*SD*)			*p*-value	*p*-value
	T1	T2	T3	T1-T2	T1-T3
**Sample (*n* =)**	921–934	673–693	531–539		
Parent’s symptoms, HSCL 10^a^	4.9 (4.6)	4.6 (4.7)	4.5 (4.4)	.002	.013
Family functioning, FAD-GF^a^	19.4 (4.8)	19.4 (4.6)	19.7 (4.7)	.35	.53
Parenting style, PBI					
Warm^b^	10.6 (2.4)	10.5 (2.3)	10.5 (2.4)	.99	.36
Protective^a^	16.9 (2.0)	17.2 (1.9)	17.2 (1.9)	.002	.002
Authoritarian^a^	12.4 (1.5)	12.6 (1.5)	12.7 (1.4)	.023	.002
Parental practices, PaRCADS(N)					
Rules & consequences^b^	24.4 (3.8)	24.7 (3.8)	24.5 (3.7)	.10	.38
Health habits^b^	17.5 (3.2)	17.4 (3.0)	17.0 (3.1)	.06	.002
Managing emotions^b^	19.3 (3.4)	20.1 (3.3)	20.1 (3.3)	.002	.002
Setting goals & dealing with problems^b^	23.7 (3.9)	24.3 (3.6)	23.9 (3.7)	.002	.12
Dealing with negative emotions^b^	28.4 (3.8)	29.4 (3.7)	29.2 (4.0)	.002	.002

*p*-values for change were Benjamini Hochberg adjusted for 20 hypotheses

^a^Higher values = less adaptive parenting, ^b^Lower values = more adaptive parenting

*HSCL-10* Hopkins symptoms checklist -10 item version, *FAD-GF* Family assessment device—general functioning subscale, *PBI* Parental bonding instrument, *PaRCADS(N)* Parenting to reduce child anxiety and depression scale (Norwegian version)

## Discussion

There were no significant differences in effects on modifiable parental factors between parents in the two conditions, five parent group sessions or a psychoeducational brochure. When inspecting the confidence intervals, variation does not entirely exclude the possibility that parents in the brochure condition have a greater reduction in symptoms between baseline (T1) and post-intervention (T2) measurements, or parental practice *Rules & consequences* between baseline (T1) and 12-month follow-up (T3). However, the narrow confidence intervals suggest that any potential difference between groups is too small to be of clinical relevance, mostly spanning below one in scales that range tenfold. Overall, our results suggest that the effect on modifiable parental factors does not rely on the delivery format of the parental component in this preventive intervention.

The lack of difference between conditions is somewhat surprising, given that the parents in the group sessions condition receive information face-to-face and interact and perform exercises with their children in a supervised setting. However, both parental components, the brochure and group sessions, are grounded in the same theoretical mechanisms of change, and aim to provide the same information. Hence, while the delivery format varies, the content remains essentially the same.

On average, the total sample of parents reported significant, however, small improvements from baseline to post-intervention, with stable results at 12-month follow-up on symptom scores measured by HSCL-10 and parental practices *Managing emotions and Dealing with negative emotions*. However, parents also reported a small negative development in parenting style, with increased scores in PBI subscales *Protective* and *Authoritarian* over time. At the 12-month follow-up, the parental practice *Health habits* scores also decreased from baseline.

The *Emotion* intervention encourages parents to help their children through behavior activation and experiments. This could have led parents to see themselves as more authoritarian, taking charge and pushing their children in difficult situations. However, most changes recorded in this study were favorable, indicating that participation in an indicative preventive CBT intervention for children with internalizing problems and their parents can potentially improve modifiable parental factors. However, these changes were minor, and the lack of a control group with no parental involvement made it difficult to draw any conclusions regarding causality. Furthermore, these relatively small levels of change indicate that the parent component of *Emotion* does not adequately address vital parental factors. The *Emotion* intervention mainly aims to teach parents what their children learn in child sessions to better support and help their children outside the intervention. Greater focus on parents and enhancing modifiable factors, such as parental symptomatology and practices like emotional management, could better equip parents to support their child, and potentially boost the impact of the *Emotion* intervention on child symptoms in the long run. The intervention focuses on parents spending positive time with their children. However, our results suggest that parenting style and communication skills are not sufficiently targeted. This could be changed through, for example, exercises and supervision in sessions to enable parents to meet their sad or anxious child more adaptively. This optimization, with a focus on modifiable parental factors linked to child internalizing problems could lead to more robust, lasting results and extend positive changes in children’s symptoms and prevent relapse for the child and other family members.

According to Schleider and Weisz’s [29] tridirectional model, parental and family factors could improve children’s internalizing problems. Likewise, the easing of children’s problems could influence parental and family factors. For instance, a shift in a child’s cognitive style could enhance the parenting style and the family’s coping abilities and communication in difficult situations. This means that both changes in parental factors brought forth by the intervention, and the documented reductions in the child’s symptoms [16] could positively have affected the family dynamic and enhanced parental factors linked to child internalizing problems, such as parental symptomatology and how parents communicate with their children about emotions and their relationships.

Our study indicates that providing written information to parents with relatively high socioeconomic status, who are aware of their children’s elevated internalizing symptom levels may be recommended for preventive interventions, such as *Emotion,* as an alternative to more resource demanding parent sessions. Furthermore, several parental factors linked to children’s internalizing problems changed during this study, providing evidence that they are malleable, and therefore could be targeted in interventions involving parents.

### Strengths and Limitations

This was a large trial with a low dropout rate (6%) during the intervention [16]. Our sample varied in terms of the sex of the parents and children, and anxious or depressive symptoms. Furthermore, rigorous data collection methods were applied in a real-life setting, with trained group leaders from local school health services, allowing for some generalizability of the results to the real world.

The participant’s children were 8–12 years old with elevated, but not necessarily clinical symptom levels, and were largely below the median age of onset of depression and anxiety disorders, other than separation anxiety or phobias [32]. Our sample mainly consisted of employed parents born in Norway, with higher education. Thus, we assumed that family and parent functioning at baseline was relatively high. The low levels of adverse parental factors could have made it difficult to detect group-level changes. Furthermore, the intervention lasted eight weeks, a short period to affect traits such as parents’ symptomatology, family dynamics, parenting style and practices. Therefore, it could be that the intervention stimulates change that is apparent only later. However, results from 12-month follow-up suggests that this was not the case in this sample.

Neither this study nor the previous effect-study of Emotion [19] included a control group with no parental involvement. Thus, we cannot conclude that the observed change in parents over time is due to parents’ participation in the intervention. Sensitivity analyses including only participants who attended three or more sessions, or had read the brochure (high adherence to the intervention) gave substantially the same results. Hence, despite varying degrees of adherence in the groups, this did not significantly affect the results.

Finally, this study relies solely on self-reported parenting behaviors, optimally one could include observations of parenting and their interaction with their children. Subscales from the PBI and PaRCADS(N) had lower internal consistency values, consistent with previous research [28, 41]. The PBI was developed to target general parenting strategies in the general population. The nil findings and relatively low internal consistency estimates suggest that the PBI is not very useful when dealing with parents of children with internalizing problems. Further, there is little research on the PaRCADS(N), thus, these results should be interpreted with care.

### Implications for Future Work

The results of the current study indicate that there is little or no change in known parental risk factors related to child anxiety and depression in this sample. Given that parental components do not seem to add to the effect of child-alone interventions, we argue that future interventions should actively target modifiable parental factors such as parent’s psychopathology, family functioning, parenting style and parental practices, and investigate whether doing so would increase the effect of combined interventions.

To enhance the effect of parent sessions in *Emotion*, one could move from a child-focused to a more parent-focused approach, where children do not attend most parent sessions. Furthermore, one could target parental factors linked to child anxiety and depression and increase intensity. Our results show that some of the parenting skills improved during the intervention, i. e. parents’ symptoms, *Managing emotions* and *Dealing with negative emotions.* Although no causal inferences can be drawn, this suggests a stronger emphasis on these topics in the parent component of *Emotion*. If children are included in parents’ sessions, the focus should have a stronger emphasis on improving the parent–child relationship, i.e. by working on communication, talking about the child’s emotional difficulties, improving trust and independence and avoiding derogatory remarks (Agerup et al., 2015). Lastly, reducing the amount of talk to include more exercises between parents might add to the effect of parent sessions [2]. Changes should then be tested and compared to a control group with no parental involvement to ensure its effectiveness.

Future research could also examine the longitudinal relationships between these parental factors and children’s symptoms in preventive interventions, such as *Emotion*. This could shed light on how these factors can hinder or promote children’s progress and the directionality of any effects. Further, this information may illuminate how, or if we should include parents in future preventive interventions.

## Conclusion

There were no differences in effects between parents in the five parent group sessions and brochure conditions in terms of parental factors linked to child anxiety and depression. This suggests that the less resource-demanding delivery format, a psychoeducational brochure, may be applied. In addition, there were small changes in parental factors in the total sample, highlighting a potential to further develop the parent component of *Emotion*, and possibly other parental components, to enhance effects on parental factors, and potentially child symptoms long term.

## Data Availability

Restrictions apply to the availability of the data that support the findings of this study due to its sensitive nature and ethical restrictions, and so are not publicly available. Data are, however, available from the authors upon reasonable request.

## Footnotes

1 The parent brochure is available upon request to the authors.
